# Self-efficacy, future expectation, and perceived stress in high school student-athletes: a cross-sectional study

**DOI:** 10.3389/fpsyg.2026.1932198

**Published:** 2026-10-12

**Authors:** Ufuk Dilekçi, Suriye Tan, Lamia Selçuk, Çetin Tan, Malik Dündar, Mustafa Karadağ, Yunus Şahinler, Selin Biçer Baikoğlu, İsmail Belli, Faik Öz, Levent Ceylan

**Affiliations:** 1 Ministry of National Education, Ankara, Türkiye; 2 Quality Management Unit, Fırat University Hospital, Elazığ, Türkiye; 3 Independent Researcher, Samsun, Türkiye; 4 Department of Physical Education and Sports, Department of Psycho-Social Fields in Sports, Faculty of Sport Sciences, Fırat University, Elazığ, Türkiye; 5 Institute of Social Sciences, Nevşehir Hacı Bektaş Veli University, Nevşehir, Türkiye; 6 Department of Coaching Education, Department of Movement and Training Sciences, Faculty of Sport Sciences, Fırat University, Elazığ, Türkiye; 7 Department of Exercise and Sport Sciences for Individuals with Disabilities, Faculty of Sport Sciences, İstanbul Gelişim University, İstanbul, Türkiye; 8 Department of Coaching Education, Department of Sport Health Sciences, Faculty of Sport Sciences, İstanbul University-Cerrahpaşa, İstanbul, Türkiye; 9 Department of Exercise and Sport Sciences, Faculty of Sport Sciences, Haliç University, İstanbul, Türkiye; 10 Red and River Digital Publishing and Trade Limited Company, Rize, Türkiye; 11 Department of Sport Management, Faculty of Sport Sciences, Hitit University, Çorum, Türkiye

**Keywords:** adolescence, future expectation, perceived coping, perceived stress, self-efficacy, student-athletes

## Abstract

**Background:**

Adolescence is a period in which beliefs about personal capability and expectations for the future develop alongside increasing academic and, for student-athletes, sporting demands. This study examined the associations among self-efficacy, future expectation, and perceived stress in high school student-athletes.

**Methods:**

This cross-sectional study included 425 high school student-athletes who completed measures of self-efficacy, future expectation, and perceived stress. Confirmatory factor analyses and multivariable regression models were used to examine domain- and component-level associations.

**Results:**

Self-efficacy dimensions were positively associated with several future-expectation domains, with social self-efficacy showing the most consistent pattern after FDR correction. Emotional self-efficacy was negatively associated with Perceived Distress, whereas no self-efficacy dimension remained significantly associated with Perceived Coping after FDR correction. Future-expectation domains were not significantly associated with either perceived-stress component in the adjusted models after FDR correction. Female gender and higher grade level were associated with greater Perceived Distress.

**Conclusion:**

Self-efficacy showed domain-specific associations with future expectation and perceived stress in high school student-athletes. Emotional self-efficacy was associated with lower Perceived Distress, whereas future-expectation domains showed no significant adjusted associations with either perceived-stress component after FDR correction. Given the cross-sectional design, these findings should be interpreted as contemporaneous associations rather than causal effects.

## 1 Introduction

Adolescence is a developmental window in which young people consolidate beliefs about their own capabilities while beginning to form concrete expectations about their future. For adolescents who also engage in organized sport, these tasks unfold alongside the demands of training, competition, and performance evaluation. Sport participation during this period has been linked to a range of psychological and social benefits and to opportunities for developing transferable life skills, yet student-athletes simultaneously contend with stressors that arise from balancing academic and athletic commitments. Indeed, exposure to such intense performance demands and competitive environments can exacerbate perceived stress and negatively affect psychological functioning if appropriate coping resources are lacking (Gerber et al., 2018; Di Fronso et al., 2022). Identifying the psychological resources associated with adaptive functioning in this group is therefore relevant to both educational and sport-psychology contexts.

Student-athletes constitute a particularly relevant subgroup for examining self-efficacy, future expectation, and perceived stress because they must manage academic and athletic demands concurrently. Research on adolescent student-athletes indicates that school- and sport-related burnout can develop in parallel and that difficulties in one domain may coexist with difficulties in the other, underscoring the importance of considering the combined academic and sporting context (Sorkkila et al., 2018). Expectations within school and sport contexts have also been associated with different burnout profiles among adolescent student-athletes, indicating that expectations represent a relevant component of their broader adjustment processes (Sorkkila et al., 2017). Perceived stress and stress reactivity are likewise salient in adolescent athletes (Britton et al., 2017), while sport-school environments involve specific challenges related to time management, injury, social pressures, and balancing multiple roles (Thompson et al., 2023). In addition, sport-related exposures may shape psychological functioning; for example, greater training load has been associated with poorer subjective well-being in youth athletes, with sleep impairment accounting for part of this relationship (Watson and Brickson, 2018). Taken together, these findings support examining self-efficacy, future expectation, and perceived stress within the specific context of student-athletes’ concurrent academic and sporting demands, while also recognizing that sport-related characteristics may contribute to variability in these associations.

Self-efficacy-an individual’s belief in their capacity to organize and execute the actions needed to manage prospective situations (Bandura, 1997)-is among the most extensively studied resources of adolescence. Self-efficacy represents a positive developmental construct that has been found to be associated with reduced risk for adolescent psychopathology and higher academic achievement across several domains. Within this broad construct, beliefs about regulating emotions have received particular attention, because the perceived capacity to manage negative affect appears especially consequential for adjustment. In a prospective study of older adolescents, perceived self-efficacy for regulating positive and negative affect was accompanied by higher efficacy to manage academic development and operated largely through other forms of behavioral efficacy in governing psychosocial functioning, including depression (Bandura et al., 2003). This conceptual emphasis is reflected in the present study, which distinguishes emotional, academic, and social self-efficacy (Muris, 2001; Telef and Karaca, 2012).

Beyond its links with current functioning, self-efficacy is theoretically and empirically connected to how adolescents anticipate their futures. Adolescents’ self-efficacy beliefs to manage emotions and interpersonal relationships have been found to promote positive expectations about the future, alongside higher self-concept and life satisfaction (Caprara et al., 2006). Intervention research points in the same direction: in a tutoring program for at-risk adolescents, increased self-efficacy was associated with increased levels of future expectations (Michael, 2019). Longitudinal work likewise situates future-oriented self-efficacy within broader developmental processes; future-oriented self-efficacy and behaviors have been shown to grow out of an emerging sense of purpose that is scaffolded by parents and school counselors across the high school years (Nunes et al., 2025). Future expectation itself refers to adolescents’ anticipations and evaluations of their personal, social, economic, and educational futures (Nurmi, 1991; Seginer, 2009). Positive future orientation has been associated with more adaptive psychological outcomes, whereas negative future thinking has been linked to anxiety and depressive experiences (Chen et al., 2021; Tang et al., 2023). Within student-athlete populations, motivation and identity are also shaped by the simultaneous pursuit of academic and sporting goals, with evidence indicating that dual-career motivation and student-athlete identity may vary according to sport- and competition-related characteristics (Lupo et al., 2017a, 2017b). While a positive future expectation is generally adaptive, intense future-oriented uncertainty can manifest as a persistent apprehension regarding adverse outcomes, often conceptualized as future anxiety (Zaleski, 1996; Geylani and Çiriş Yıldız, 2022). On this basis, self-efficacy is expected to be positively associated with future expectation among student-athletes.

Perceived stress reflects the degree to which individuals appraise the demands in their lives as exceeding their resources to cope (Cohen et al., 1983; Lazarus and Folkman, 1984). Within the transactional model, appraisal is central, and self-efficacy is a core appraisal-relevant resource. Individuals with high self-efficacy tend to draw on internal resources to confront life’s changes and show stronger stress-management capacity, whereas those with lower self-efficacy more often perceive themselves as unable to manage stressors and adopt passive coping. Consistent with this, academic self-efficacy has been found to partially mediate the longitudinal association between academic stress and psychological distress among upper-secondary students (Kristensen et al., 2023). Emotion-related efficacy has received particular attention in this context, in line with the prominence of the emotional component noted above. These patterns lead to the expectation that self-efficacy-particularly its emotional facet-will be negatively associated with perceived stress.

The association between future expectation and stress, however, is less straightforward, and the literature is genuinely mixed. On one hand, a positive future orientation may buffer distress: in university students during the COVID-19 outbreak, future orientation lowered anxiety through optimization in primary and secondary control and through resilience (Chen et al., 2021). On the other hand, stronger future orientation has sometimes been associated with greater, not lesser, anxiety. In a large study of school students grounded in control-value theory, future orientation significantly and positively predicted academic anxiety, a finding the authors noted was inconsistent with some prior research (Li et al., 2024). This divergence indicates that the direction of the future expectation–stress association should be treated as an open empirical question rather than assumed in advance, and it is examined as such in the present study.

Taken together, prior research has established that self-efficacy is associated with both future expectation and stress, whereas the association between future expectation and stress remains less consistent. However, these constructs have rarely been examined simultaneously among adolescent student-athletes, who face concurrent academic and sporting demands. Accordingly, the present cross-sectional study examined the domain-specific associations among self-efficacy, future expectation, and perceived stress in high school student-athletes. Specifically, we examined whether emotional, academic, and social self-efficacy were associated with economic, social, educational, and personal/vocational future expectations and whether self-efficacy dimensions and future-expectation domains were independently associated with perceived distress and perceived coping. Given the cross-sectional design, these associations were interpreted as contemporaneous rather than temporal or causal relationships. Based on this theoretical and empirical background, we tested the following hypotheses: H1: Self-efficacy dimensions are positively associated with future-expectation domains among high school student-athletes. H2: Self-efficacy dimensions are associated with lower Perceived Distress and higher Perceived Coping among high school student-athletes. H3: Future-expectation domains are associated with perceived-stress components, with the direction of these associations examined empirically rather than specified *a priori*.

## 2 Methods

### 2.1 Participants

Participants were recruited between September and December 2024 from public high schools located in six provinces of Türkiye (Ankara, Elazığ, Nevşehir, İstanbul, Trabzon, and Çorum). Recruitment was coordinated by members of the research team based in or affiliated with institutions in these regions, in collaboration with school administrators, physical education teachers, and coaches. A convenience-sampling approach was used across participating schools. Students were eligible to participate if they were enrolled in grades 9–12, were actively involved in organized sport, and had participated in structured sport activities for at least 6 months. Organized sport participation was operationally defined as regular involvement in coach-supervised school- or club-based sport at least twice per week. Students who did not meet these eligibility criteria or who submitted incomplete questionnaires were excluded from the analytic sample. Participation was voluntary, and students who declined consent were not enrolled in the study. A total of 468 students were initially approached. After eligibility screening, 451 students met the inclusion criteria. Of these, 438 provided consent and completed the study procedures. Thirteen questionnaires were excluded because of incomplete data, resulting in a final analytic sample of 425 participants. Recruitment was facilitated through the Ministry of National Education and participating public high schools in the six provinces. Because school-level identifiers were not retained in the analytic dataset, the total number of participating schools and potential within-school clustering could not be examined directly. Team-level clustering could likewise not be evaluated because team identifiers were not retained. Participant demographic and sport-related characteristics are presented in Table 1.

**Table 1:** Demographic characteristics of the participants.

Variable	Category	n	%
Gender	Male	267	62.8
	Female	158	37.2
Grade	9th grade	114	26.8
	10th grade	110	25.9
	11th grade	104	24.5
	12th grade	97	22.8
Sport type	Individual	143	33.6
	Team	282	66.4
Mother’s education	Primary	13	3.1
	Secondary	68	16.0
	High school	257	60.5
	University	87	20.5
Father’s education	Primary	41	9.6
	Secondary	33	7.8
	High school	213	50.1
	University	138	32.5
Perceived family income	Low	122	28.7
	Middle	217	51.1
	High	86	20.2
Own study room	Yes	316	74.4
	No	109	25.6
Licensed athlete in family	Yes	52	12.2
	No	67	15.8
	Do not know	306	72.0

*N* = 425.

### 2.2 Ethics approval and consent to participate

This study was approved by the Istanbul Rumeli University Ethics Committee (Approval No: E-53938333-050-41073; Date: 02 August 2024). All procedures performed in this study involving human participants were conducted in accordance with the ethical standards of the institutional research committee and with the 1964 Declaration of Helsinki and its later amendments or comparable ethical standards. Participation was voluntary, and written informed consent was obtained from all participants and, where required, from their legal guardians prior to data collection.

### 2.3 Measurement instruments

#### 2.3.1 Adolescent future expectation scale

Future expectation was assessed using the Adolescent Future Expectation Scale adapted into Turkish by Tuncer (2011), which measures adolescents’ anticipations regarding their forthcoming life circumstances. The scale consists of 33 items rated on a 5-point Likert-type response format and yields four sub-dimensions: Economic, Social, Educational, and Personal/Vocational future expectation. Psychometric analyses of the scale in the present sample are reported in the Results section.

#### 2.3.2 Self-efficacy scale for children

Self-efficacy was measured using the Self-Efficacy Scale for Children, originally developed by Muris (2001) and adapted into Turkish by Telef and Karaca (2012). The scale assesses an individual’s perceived capacity to manage emotional, academic, and social demands, and comprises 21 items rated on a 5-point Likert-type response format with a three-factor structure (Emotional, Academic, and Social self-efficacy). Psychometric analyses of the scale in the present sample are reported in the Results section.

#### 2.3.3 Perceived stress scale (PSS-14)

Perceived stress was assessed using the 14-item Perceived Stress Scale, developed by Cohen et al. (1983) and adapted into Turkish by Eskin et al. (2013). Items are rated on a 5-point response format (0 = never to 4 = very often), with seven reverse-scored items, and yield two components-Perceived Coping and Perceived Distress-together with a total score; higher total scores indicate greater perceived stress. For component-level analyses, the Perceived Coping items were scored in their original positive direction so that higher component scores reflected greater perceived coping capacity; conventional reverse scoring was retained when calculating the overall PSS total score. Psychometric analyses of the scale in the present sample are reported in the Results section.

### 2.4 Statistical analysis

Analyses were conducted using IBM SPSS Statistics 27 (IBM Corp., 2020) and the lavaan package in R (Rosseel, 2012). Data management and supplementary descriptive profiling were supported by the R statistical environment (R Core Team, 2020), utilizing the *psych* package (Revelle, 2024) and *dplyr*/*readxl* packages (Wickham et al., 2023; Wickham and Bryan, 2023). Furthermore, distributional properties and multivariate assumptions were examined in line with the guidelines provided by Tabachnick and Fidell (2013). Prior to analysis, the data were screened for missing values and outliers. The psychometric properties of each scale were examined through exploratory factor analysis using maximum likelihood extraction with oblimin rotation; sampling adequacy was evaluated with the Kaiser–Meyer–Olkin (KMO) measure and Bartlett’s test of sphericity (Field, 2013). Internal consistency was estimated with Cronbach’s alpha (Cronbach, 1951), while composite reliability (CR) and average variance extracted (AVE) were examined as additional indicators of measurement quality, with AVE interpreted against the conventional 0.50 criterion (Fornell and Larcker, 1981; Hair et al., 2019). To further evaluate the measurement structure of the study instruments before testing the structural associations, confirmatory factor analyses (CFA) were conducted for the Adolescent Future Expectation Scale, the Self-Efficacy Scale for Children, and the Perceived Stress Scale. The items were treated as ordered categorical indicators, and the CFA models were estimated using the robust weighted least squares estimator (WLSMV). Model fit was evaluated using the comparative fit index (CFI), Tucker–Lewis index (TLI), root mean square error of approximation (RMSEA), and standardized root mean square residual (SRMR; Hu and Bentler, 1999). Standardized factor loadings, composite reliability (CR), and average variance extracted (AVE) were additionally examined to assess measurement quality and convergent validity. CFA results were interpreted jointly with the reliability and convergent-validity evidence rather than solely on the basis of global model-fit indices. On the basis of the confirmatory measurement results, the multidimensional structure of the study constructs was retained in the primary inferential analyses. Accordingly, the four future-expectation domains and the two perceived-stress components were examined separately rather than being represented solely by their respective total scores. Emotional, Academic, and Social self-efficacy were likewise examined as distinct dimensions in multivariable analyses, and the overall self-efficacy score was not entered simultaneously with its constituent subscales. Total scale scores were retained for descriptive and bivariate contextual purposes only. This approach was adopted to avoid obscuring domain-specific associations and to reduce conceptual and mathematical overlap between total scores and their component dimensions. Because both the exploratory and confirmatory factor analyses were conducted in the same sample, the measurement findings are interpreted as sample-specific evidence rather than as independent cross-validation; this limitation is acknowledged in the Discussion.

Distributional properties were initially evaluated using skewness and kurtosis coefficients, with values within ±2 considered acceptable for descriptive purposes (George and Mallery, 2010). Regression diagnostics were subsequently evaluated at the model level, including homoscedasticity, multicollinearity, residual distribution, and influential observations. Linear associations among variables were examined using Pearson correlation analysis. Adjusted associations were examined using multivariable linear regression models. For the future-expectation outcomes, Emotional, Academic, and Social self-efficacy were entered simultaneously as predictors in separate models for the Economic, Social, Educational, and Personal/Vocational domains. For the perceived-stress outcomes, the three self-efficacy dimensions and four future-expectation domains were entered simultaneously, with gender and grade level included as covariates, in separate models for Perceived Distress and Perceived Coping. For each regression model, unstandardized coefficients (B), standard errors (SE), standardized coefficients (β), 95% confidence intervals (CI), *p* values, and model-level statistics including R^2^, adjusted R^2^, and the omnibus F statistic were reported. Multicollinearity was assessed using variance inflation factors and tolerance values. To account for multiple testing, the Benjamini–Hochberg false discovery rate (FDR) correction was applied separately to the 12 regression coefficient tests in Table 2 and the 18 regression coefficient tests in Table 3. FDR-adjusted q values < 0.05 were considered statistically significant.

**Table 2:** Multivariable regression models predicting future-expectation domains from self-efficacy dimensions.

Outcome	Predictor	B	SE	β	95% CI	*p*	q
Economic expectation	Emotional self-efficacy	0.108	0.073	0.100	[−0.035, 0.250]	0.139	0.167
	Academic self-efficacy	0.098	0.069	0.082	[−0.038, 0.234]	0.158	0.172
	Social self-efficacy	0.422	0.075	0.318	[0.274, 0.570]	< 0.001	< 0.001
Social expectation	Emotional self-efficacy	0.068	0.059	0.075	[−0.048, 0.183]	0.249	0.249
	Academic self-efficacy	0.094	0.056	0.094	[−0.016, 0.204]	0.093	0.127
	Social self-efficacy	0.428	0.061	0.386	[0.309, 0.548]	< 0.001	< 0.001
Educational expectation	Emotional self-efficacy	0.116	0.070	0.114	[−0.021, 0.254]	0.096	0.127
	Academic self-efficacy	0.179	0.066	0.158	[0.049, 0.310]	0.007	0.017
	Social self-efficacy	0.276	0.072	0.219	[0.134, 0.418]	< 0.001	0.001
Personal/Vocational expectation	Emotional self-efficacy	0.139	0.068	0.142	[0.005, 0.272]	0.042	0.072
	Academic self-efficacy	0.135	0.065	0.124	[0.008, 0.262]	0.038	0.072
	Social self-efficacy	0.214	0.070	0.177	[0.076, 0.352]	0.002	0.007

Model statistics. Economic expectation: R^2^ = 0.190, adjusted R^2^ = 0.184, F(3, 421) = 32.92, *p* < 0.001. Social expectation: R^2^ = 0.240, adjusted R^2^ = 0.235, F(3, 421) = 44.32, *p* < 0.001. Educational expectation: R^2^ = 0.170, adjusted R^2^ = 0.164, F(3, 421) = 28.74, *p* < 0.001. Personal/Vocational expectation: R^2^ = 0.140, adjusted R^2^ = 0.134, F(3, 421) = 22.84, *p* < 0.001. *N* = 425. B, unstandardized regression coefficient; SE, standard error; β, standardized regression coefficient; CI, confidence interval. Emotional, academic, and social self-efficacy were entered simultaneously in each model. Benjamini–Hochberg false discovery rate correction was applied across the 12 coefficient tests. Adjusted q values < 0.05 were considered statistically significant.

**Table 3:** Multivariable regression models predicting perceived-stress components.

Outcome	Predictor	B	SE	β	95% CI	*p*	q
Perceived distress	Emotional self-efficacy	−2.024	0.408	−0.333	[−2.825, −1.222]	< 0.001	< 0.001
	Academic self-efficacy	−0.653	0.391	−0.097	[−1.422, 0.116]	0.096	0.216
	Social self-efficacy	0.322	0.461	0.043	[−0.583, 1.227]	0.485	0.502
	Economic expectation	−0.182	0.271	−0.032	[−0.714, 0.350]	0.502	0.502
	Social expectation	−0.435	0.339	−0.064	[−1.101, 0.231]	0.200	0.354
	Educational expectation	0.639	0.283	0.107	[0.082, 1.195]	0.025	0.089
	Personal/Vocational expectation	0.534	0.291	0.086	[−0.038, 1.106]	0.067	0.192
	Gender	2.914	0.493	0.258	[1.946, 3.883]	< 0.001	< 0.001
	Grade	0.738	0.214	0.150	[0.318, 1.159]	< 0.001	0.004
Perceived coping	Emotional self-efficacy	0.927	0.410	0.159	[0.122, 1.733]	0.024	0.089
	Academic self-efficacy	0.703	0.393	0.108	[−0.070, 1.475]	0.075	0.192
	Social self-efficacy	0.520	0.463	0.072	[−0.389, 1.429]	0.262	0.362
	Economic expectation	0.274	0.272	0.051	[−0.261, 0.808]	0.314	0.404
	Social expectation	0.422	0.340	0.065	[−0.248, 1.091]	0.216	0.354
	Educational expectation	0.206	0.284	0.036	[−0.353, 0.765]	0.469	0.502
	Personal/Vocational expectation	0.345	0.292	0.058	[−0.229, 0.919]	0.238	0.358
	Gender	−0.627	0.495	−0.058	[−1.600, 0.346]	0.206	0.354
	Grade	−0.204	0.215	−0.043	[−0.627, 0.218]	0.342	0.411

Model statistics. Perceived Distress: R^2^ = 0.230, adjusted R^2^ = 0.213, F(9, 415) = 13.77, *p* < 0.001. Perceived Coping: R^2^ = 0.160, adjusted R^2^ = 0.142, F(9, 415) = 8.78, *p* < 0.001. *N* = 425. B, unstandardized regression coefficient; SE, standard error; β, standardized regression coefficient; CI, confidence interval. Emotional, academic, and social self-efficacy; economic, social, educational, and personal/vocational future expectation; gender; and grade were entered simultaneously in each model. Gender was coded 0 = male and 1 = female. Grade level was coded from 9 to 12 and modeled as a linear covariate. Benjamini–Hochberg false discovery rate correction was applied across the 18 coefficient tests. FDR-adjusted q values < 0.05 were considered statistically significant.

## 3 Results

### 3.1 Exploratory measurement analyses

Exploratory factor analysis of the Adolescent Future Expectation Scale yielded a four-factor solution consistent with the intended structure and accounted for 57.9% of the total variance (KMO = 0.804; Bartlett’s χ^2^(528) = 14689.88, *p* < 0.001). Internal consistency was high across the four subscales, with Cronbach’s alpha values ranging from 0.894 to 0.922. Detailed factor loadings and internal-consistency coefficients are presented in Table 4.

**Table 4:** Exploratory factor analysis and internal consistency of the adolescent future expectation scale.

Item	F1 economic	F2 social	F3 educational	F4 personal/vocational
AFES1 – Living conditions	0.594			
AFES2 – Economic conditions	0.900			
AFES3 – Living standards	0.846			
AFES4 – Welfare level	0.893			
AFES5 – Job opportunities	0.611			
AFES6 – Country living conditions	0.595			
AFES7 – City living conditions	0.594			
AFES8 – Rapid problem-solving	0.488			
AFES9 – Fewer problems		0.551		
AFES10 – People’s happiness		0.623		
AFES11 – Peaceful democratic society		0.705		
AFES12 – Human relations		0.511		
AFES13 – Rule of law		0.745		
AFES14 – Tolerant people		0.831		
AFES15 – Respect for differences		0.515		
AFES16 – Family/kinship relations		0.378		
AFES17 – School facilities			0.723	
AFES18 – Qualified teachers			0.363	
AFES19 – Students’ conditions			0.813	
AFES20 – Qualified students			0.730	
AFES21 – School education quality			0.678	
AFES22 – Family involvement			0.358	
AFES23 – School administrators’ competence			0.502	
AFES24 – Contribution to profession				0.894
AFES25 – Career advancement				0.850
AFES26 – Responsible individual				0.807
AFES27 – Enterprising individual				0.556
AFES28 – Independent individual				0.488
AFES29 – Forming a happy family				0.683
AFES30 – Self-management				0.864
AFES31 – Meaningfulness of life				0.606
AFES32 – Useful to society				0.576
AFES33 – Respected social position				0.837
Cronbach α	0.912	0.894	0.902	0.922

Extraction method: maximum likelihood; rotation: oblimin. Loadings are shown only for the theoretically assigned factor. All retained primary factor loadings exceeded 0.35.

Exploratory factor analysis of the Self-Efficacy Scale for Children yielded a three-factor solution consistent with the intended structure and accounted for 46.9% of the total variance (KMO = 0.796; Bartlett’s χ^2^(210) = 4864.66, *p* < 0.001). The overall scale demonstrated strong internal consistency (Cronbach’s alpha = 0.907), with subscale alpha values ranging from 0.784 to 0.843. Detailed factor loadings and internal-consistency coefficients are presented in Table 5.

**Table 5:** Exploratory factor analysis and internal consistency of the Self-Efficacy Scale for Children.

Item	F1 emotional	F2 academic	F3 social
SE2 – Cheering oneself up	0.592		
SE4 – Calming down	0.625		
SE8 – Controlling temper	0.602		
SE11 – Controlling emotions	0.522		
SE14 – Encouraging oneself	0.346		
SE19 – Suppressing distressing thoughts	0.610		
SE21 – Not worrying	0.739		
SE3 – Studying		0.506	
SE6 – Exam preparation		0.390	
SE9 – Completing homework		0.530	
SE12 – Concentrating		0.850	
SE15 – Understanding lessons		0.350	
SE17 – Pleasing the family		0.497	
SE20 – Passing exams		0.496	
SE1 – Expressing opinions			0.351
SE5 – Making friends			0.575
SE7 – Chatting with strangers			0.713
SE10 – Working cooperatively			0.729
SE13 – Expressing dislikes			0.388
SE16 – Telling a story			0.595
SE18 – Maintaining friendships			0.644
Cronbach α	0.843	0.807	0.784

Extraction method: maximum likelihood; rotation: oblimin. Loadings are shown only for the theoretically assigned factor. Overall scale Cronbach’s α = 0.907.

Exploratory factor analysis of the Perceived Stress Scale yielded a two-factor solution consistent with the intended structure and accounted for 43.7% of the total variance (KMO = 0.724; Bartlett’s χ^2^(91) = 2653.70, *p* < 0.001). Internal consistency was acceptable for Perceived Coping (Cronbach’s alpha = 0.841), Perceived Distress (Cronbach’s alpha = 0.799), and the overall scale (Cronbach’s alpha = 0.778). Detailed factor loadings and internal-consistency coefficients are presented in Table 6.

**Table 6:** Exploratory factor analysis and internal consistency of the Perceived Stress Scale (PSS-14).

Item	F1 perceived coping	F2 perceived distress
PSS4 (R) – Coping successfully	0.735	
PSS5 (R) – Coping with changes	0.550	
PSS6 (R) – Coping with personal problems	0.548	
PSS7 (R) – Things going your way	0.900	
PSS9 (R) – Controlling irritations	0.798	
PSS10 (R) – Being on top of things	0.542	
PSS13 (R) – Controlling time	0.475	
PSS1 – Upset by the unexpected		0.595
PSS2 – Unable to control important things		0.477
PSS3 – Feeling nervous and stressed		0.746
PSS8 – Unable to cope with everything		0.688
PSS11 – Angered by things outside control		0.620
PSS12 – Thinking of things to accomplish		0.592
PSS14 – Difficulties piling up		0.536
Cronbach α	0.841	0.799

Extraction method: maximum likelihood; rotation: oblimin. Loadings are shown only for the theoretically assigned factor. Items marked (R) are conventionally reverse-scored when calculating the PSS total score; for component-level analyses, these items were scored in their original positive direction so that higher scores indicated greater perceived coping capacity. Overall scale Cronbach’s α = 0.778. Inter-factor correlation: *r* = −0.043.

### 3.2 Confirmatory measurement models

Confirmatory factor analyses indicated acceptable global fit for the hypothesized multidimensional structures of the three study instruments. The four-factor model of the Adolescent Future Expectation Scale showed acceptable fit to the data (CFI = 0.928, TLI = 0.920, RMSEA = 0.052, SRMR = 0.059). The three-factor model of the Self-Efficacy Scale for Children also demonstrated acceptable fit (CFI = 0.941, TLI = 0.933, RMSEA = 0.050, SRMR = 0.051), as did the two-factor model of the Perceived Stress Scale (CFI = 0.946, TLI = 0.936, RMSEA = 0.051, SRMR = 0.048). However, convergent validity remained limited for several subscales, as indicated by AVE values below the conventional 0.50 criterion, with the Educational future-expectation dimension showing the weakest convergent validity. Therefore, although the overall factor structures were supported at the global-fit level, subscale-level results were interpreted cautiously and were not treated as evidence of uniformly strong measurement quality. Detailed reliability and convergent-validity results are presented in Table 7. Standardized factor loadings were also examined as part of the confirmatory measurement assessment. Given the suboptimal convergent-validity evidence for several dimensions, interpretation of the latent constructs was based on the combined pattern of factor loadings, composite reliability, AVE, and global model-fit indices rather than on fit statistics alone.

**Table 7:** Confirmatory factor analysis and convergent validity results.

Scale/Subscale	Factor structure	CFI	TLI	RMSEA	SRMR	CR	AVE
Adolescent future expectation scale	Four correlated factors	0.928	0.920	0.052	0.059		
Economic						0.863	0.447
Social						0.834	0.393
Educational						0.808	0.383
Personal/Vocational						0.909	0.504
Self-efficacy scale for children	Three correlated factors	0.941	0.933	0.050	0.051		
Emotional						0.799	0.365
Academic						0.765	0.322
Social						0.805	0.375
Perceived stress scale	Two correlated factors	0.946	0.936	0.051	0.048		
Perceived Coping						0.832	0.421
Perceived Distress						0.809	0.380

CFI, comparative fit index; TLI, Tucker–Lewis index; RMSEA, root mean square error of approximation; SRMR, standardized root mean square residual; CR, composite reliability; AVE, average variance extracted. AVE was below 0.50 for most subscales; therefore, subscale-level findings were interpreted cautiously.

### 3.3 Descriptive statistics and correlations

Descriptive statistics for the study variables are presented in Table 8. Skewness and kurtosis values for all variables fell within ±2 and were considered acceptable for descriptive purposes. Among the future-expectation domains, Personal/Vocational expectation had the highest mean (M = 3.76, SD = 0.88), whereas Social expectation had the lowest mean (M = 2.64, SD = 0.81). Among the self-efficacy dimensions, Social self-efficacy had the highest mean (M = 3.55, SD = 0.73). The overall PSS score had a mean of 27.42 (SD = 7.75).

**Table 8:** Descriptive statistics for the study variables.

Variable	M	SD	Skewness	Kurtosis
AFES - Economic future	2.83	0.97	0.19	−0.28
AFES - Social future	2.64	0.81	−0.17	0.22
AFES - Educational future	2.89	0.92	0.00	0.13
AFES - Personal/Vocational	3.76	0.88	−0.09	−0.31
AFES - Total	3.08	0.57	0.23	−0.50
SE - Emotional	3.28	0.90	0.13	−0.42
SE - Academic	3.16	0.81	0.07	−0.28
SE - Social	3.55	0.73	0.07	−0.42
SE - Total	3.33	0.69	0.14	−0.60
PSS - Perceived Distress	13.21	5.47	0.08	−0.25
PSS - Perceived Coping	13.79	5.26	0.18	−0.08
PSS - Total	27.42	7.75	−0.05	0.07

*N =* 425. AFES, Adolescent Future Expectation Scale; SE, Self-Efficacy; PSS, Perceived Stress Scale. Higher Perceived Coping scores indicate greater perceived coping capacity. Skewness and kurtosis values within ±2 were considered acceptable for descriptive purposes.

Pearson correlations are reported in Table 9. The future-expectation domains were positively correlated with one another, with the strongest association observed between Social and Educational expectation (*r* = 0.27, *p* < 0.001). The self-efficacy dimensions were also positively correlated, with the strongest association between Emotional and Academic self-efficacy (*r* = 0.65, *p* < 0.001). Perceived Distress was negatively correlated with Emotional (*r* = −0.34, *p* < 0.001), Academic (*r* = −0.29, *p* < 0.001), and Social self-efficacy (*r* = −0.20, *p* < 0.001), as well as with Economic (*r* = −0.15, *p* < 0.01) and Social future expectation (*r* = −0.15, *p* < 0.01). Perceived Coping was positively correlated with all three self-efficacy dimensions (*r* = 0.30–0.35, all *p* < 0.001) and all four future-expectation domains (*r* = 0.19–0.23, all *p* < 0.001). The PSS total score was negatively correlated with all self-efficacy dimensions (*r* = −0.35 to −0.48, all *p* < 0.001) and all future-expectation domains (*r* = −0.15 to −0.26, all *p* < 0.01). Perceived Distress and Perceived Coping were not significantly correlated (*r* = −0.04, *p* = 0.377).

**Table 9:** Pearson correlation matrix (diagonal: Cronbach’s alpha).

Variable	1	2	3	4	5	6	7	8	9	10
1. AFES-Economic	(0.91)									
2. AFES-Social	0.25***	(0.89)								
3. AFES-Educational	0.21***	0.27***	(0.90)							
4. AFES-Personal/Vocational	0.15**	0.24***	0.18***	(0.92)						
5. SE-Emotional	0.35***	0.38***	0.35***	0.33***	(0.84)					
6. SE-Academic	0.28***	0.31***	0.32***	0.29***	0.65***	(0.81)				
7. SE-Social	0.42***	0.47***	0.36***	0.32***	0.63***	0.42***	(0.78)			
8. PSS-Perceived Distress	−0.15**	−0.15**	−0.06	−0.03	−0.34***	−0.29***	−0.20***	(0.80)		
9. PSS-Perceived Coping	0.20***	0.23***	0.20***	0.19***	0.35***	0.31***	0.30***	−0.04	(0.84)	
10. PSS-Total	−0.24***	−0.26***	−0.17***	−0.15**	−0.48***	−0.41***	−0.35***	0.73***	−0.71***	(0.78)

*N* = 425. Diagonal values in parentheses are Cronbach’s alpha coefficients. Higher Perceived Coping scores indicate greater perceived coping capacity. Correlation analyses were treated as descriptive and bivariate contextual analyses and were not included in the FDR-adjusted primary regression test families. *p < 0.05. **p < 0.01. ***p < 0.001.

### 3.4 Adjusted associations: self-efficacy, future expectation, and perceived stress (testing H1, H2, and H3)

Primary inferential analyses were conducted at the domain/component level, consistent with the multidimensional measurement structure of the study constructs. Associations involving the four future-expectation domains, the three self-efficacy dimensions, and the two perceived-stress components were examined separately to avoid reliance on potentially heterogeneous total scores. Total-score analyses were retained only for descriptive and bivariate contextual purposes. Multicollinearity diagnostics indicated no problematic collinearity among the predictors, with variance inflation factors remaining below 3 and tolerance values above 0.30 across the regression models. As shown in Table 2, the multivariable regression models predicting the four future-expectation domains were statistically significant, explaining 19% of the variance in Economic expectation, 24% in Social expectation, 17% in Educational expectation, and 14% in Personal/Vocational expectation. Social self-efficacy was positively associated with Economic (*β* = 0.318, *p* < 0.001, q < 0.001), Social (*β* = 0.386, *p* < 0.001, q < 0.001), Educational (*β* = 0.219, *p* < 0.001, q = 0.001), and Personal/Vocational expectations (*β* = 0.177, *p* = 0.002, q = 0.007). Academic self-efficacy was positively associated with Educational expectation (*β* = 0.158, *p* = 0.007, q = 0.017), whereas its associations with Economic, Social, and Personal/Vocational expectations did not remain statistically significant after FDR correction. Emotional self-efficacy was not significantly associated with Economic, Social, or Educational expectation, and its nominal association with Personal/Vocational expectation (*β* = 0.142, *p* = 0.042) did not remain statistically significant after FDR correction (q = 0.072). Overall, H1 was partially supported at the domain level, with social self-efficacy showing the most consistent pattern of positive adjusted associations.

As shown in Table 3, the multivariable model for Perceived Distress was statistically significant, *F*(9, 415) = 13.77, *p* < 0.001, explaining 23.0% of the variance (adjusted R^2^ = 0.213). Emotional self-efficacy was negatively associated with Perceived Distress (*β* = −0.333, *p* < 0.001, q < 0.001). Female gender (*β* = 0.258, *p* < 0.001, q < 0.001) and higher grade level (*β* = 0.150, *p* < 0.001, q = 0.004) were positively associated with Perceived Distress. Educational future expectation showed a nominal positive association with Perceived Distress (*β* = 0.107, *p* = 0.025), but this association did not remain statistically significant after FDR correction (q = 0.089). No other predictors were significantly associated with Perceived Distress after FDR correction. The Perceived Coping model was also statistically significant, *F*(9, 415) = 8.78, *p* < 0.001, explaining 16.0% of the variance (adjusted R^2^ = 0.142). Although emotional self-efficacy showed a nominal positive association with Perceived Coping (*β* = 0.159, *p* = 0.024), it did not remain statistically significant after FDR correction (q = 0.089). None of the remaining predictors were significantly associated with Perceived Coping after FDR correction. Overall, H2 received partial support through the association between emotional self-efficacy and Perceived Distress, whereas H3 was not supported in the FDR-adjusted domain-level models.

## 4 Discussion

This cross-sectional study examined the associations among self-efficacy, future expectation, and perceived stress in high school student-athletes. Self-efficacy dimensions were positively associated with several future-expectation domains, with social self-efficacy showing the most consistent pattern of associations. In the perceived-stress models, emotional self-efficacy was negatively associated with Perceived Distress after adjustment for the other predictors and FDR correction. In contrast, no self-efficacy dimension remained significantly associated with Perceived Coping after FDR correction, and none of the future-expectation domains showed a significant independent association with either perceived-stress component. Thus, the adjusted models did not provide evidence that future-expectation domains were independently associated with either greater or lower perceived stress. Given the cross-sectional and self-report design, the results should be interpreted as contemporaneous associations rather than temporal or causal mechanisms.

### 4.1 Self-efficacy and future expectation

H1 was partially supported, as self-efficacy dimensions were positively associated with several, but not all, future-expectation domains. Social self-efficacy showed the most consistent pattern, remaining positively associated with Economic, Social, Educational, and Personal/Vocational expectations after FDR correction. Academic self-efficacy was positively associated only with Educational expectation after correction, whereas its associations with the other future-expectation domains were not statistically significant. Emotional self-efficacy did not show significant adjusted associations with Economic, Social, or Educational expectation, and its nominal association with Personal/Vocational expectation did not remain significant after FDR correction. Taken together, these findings suggest that the association between self-efficacy and future expectation was domain-specific rather than uniform across self-efficacy dimensions.

### 4.2 Self-efficacy and perceived stress

H2 was partially supported. Emotional self-efficacy was negatively associated with Perceived Distress after adjustment for the other predictors and FDR correction. In contrast, no self-efficacy dimension remained significantly associated with Perceived Coping after FDR correction. This pattern is consistent with the transactional model of stress, in which appraisal of coping resources is central (Lazarus and Folkman, 1984), and with research on emotion-related efficacy. Bandura et al. (2003) linked efficacy for regulating affect to better psychosocial functioning, while Caprara et al. (2008) associated regulatory emotional self-efficacy with lower anxiety and depressive problems. Emotional self-efficacy may therefore be particularly relevant to perceived distress among adolescent student-athletes, although the present analyses do not establish that it is statistically stronger than the academic or social dimensions. This interpretation is also consistent with evidence that internal psychological resources may support more adaptive responses to stress in demanding athletic and academic contexts (Gerber et al., 2018; Hu et al., 2015). The absence of FDR-adjusted associations with Perceived Coping, however, suggests that coping-related findings should be interpreted cautiously and should not be taken as evidence of a robust independent association in the present sample.

### 4.3 Future expectation and perceived stress

H3 was not supported in the adjusted domain/component-level analyses. None of the future-expectation domains remained significantly associated with either Perceived Distress or Perceived Coping after adjustment for self-efficacy dimensions, gender, and grade level and after FDR correction. Thus, the present adjusted models did not provide evidence that specific future-expectation domains were independently associated with either greater or lower perceived stress. This finding is consistent with a mixed literature, with some studies linking future-oriented concerns to career uncertainty and demanding personal goals (Dalmış et al., 2025; Kula and Saraç, 2016), whereas others associate a more positive future orientation with lower psychological distress (Chen et al., 2021; Tang et al., 2023). These contrasting findings suggest that associations between future orientation and stress may depend on how future-related constructs are conceptualized and measured, as well as on the population and context under study. From a broader interpretive perspective, psychological resources such as resilience and self-efficacy may help adolescents manage future-related uncertainty and maintain a more positive future outlook rather than experiencing such uncertainty primarily as a source of stress (Hu et al., 2015; Kula and Saraç, 2016). Longitudinal research is needed to determine whether specific future-expectation domains prospectively relate to perceived stress in student-athletes.

### 4.4 Gender and grade-level associations

In the adjusted model, female gender and higher grade level were both associated with greater Perceived Distress after FDR correction. The gender-related pattern is consistent with broader evidence indicating higher levels of school-related stress among adolescent girls than boys (García-Moya et al., 2025) and with documented gender differences in regulatory emotional self-efficacy (Caprara et al., 2008). The positive association between grade level and Perceived Distress may reflect greater academic and transition-related demands during the later years of high school; however, this interpretation remains tentative because these contextual demands were not measured directly in the present study. Accordingly, these demographic findings should be interpreted as adjusted cross-sectional associations rather than as evidence that gender or grade level causes differences in perceived distress.

## 5 Originality

The present study contributes to the literature by examining self-efficacy, future expectation, and perceived stress jointly in adolescent student-athletes, a population managing concurrent academic and sporting demands. A further contribution is the use of a domain/component-level analytical approach, which revealed that associations varied across specific dimensions rather than following a uniform pattern. Social self-efficacy showed the most consistent positive associations with future-expectation domains, whereas emotional self-efficacy was associated with lower Perceived Distress in the adjusted model after FDR correction. In contrast, no self-efficacy dimension remained significantly associated with Perceived Coping after FDR correction, and none of the future-expectation domains showed significant adjusted associations with either perceived-stress component. These findings highlight the value of examining self-efficacy, future expectation, and perceived stress multidimensionally and caution against assuming a uniform association between future expectation and perceived stress in student-athletes.

### 5.1 Limitations and directions for future research

The current study has several methodological constraints. First, the cross-sectional, single-time-point design does not permit temporal or causal inference, while reliance on self-report measures may increase the risk of common method variance. Accordingly, the observed associations should be interpreted as contemporaneous rather than as evidence of temporal or causal mechanisms. Longitudinal designs are needed to clarify the temporal ordering among self-efficacy, future expectation, and perceived stress. Second, because both the exploratory and confirmatory factor analyses were conducted in the same sample (N = 425), the measurement findings constitute sample-specific evidence rather than independent cross-validation; future studies should employ split-sample or independent-sample validation. Third, AVE values were below the conventional 0.50 threshold for several future-expectation dimensions and for all three self-efficacy dimensions, suggesting limited convergent validity at the sub-dimension level; findings involving these dimensions should therefore be interpreted cautiously. Fourth, the convenience sampling strategy and the predominance of male (62.8%) and team-sport (66.4%) participants may limit generalizability to more balanced or individual-sport samples. In addition, school- and team-level identifiers were not retained, preventing direct evaluation of potential clustering of participants within schools or teams. Finally, although sport type was recorded, other potentially relevant sport-specific characteristics, including competition level, training volume, years of participation, and injury history, were not assessed. This limitation may be particularly relevant because previous research has shown that competition level is associated with student-athletes’ sport and dual-career motivation as well as with aspects of their academic and athletic identity (Lupo et al., 2017a, 2017b). Future studies should therefore examine competition level alongside training volume, years of participation, and injury history when investigating variation in the associations among self-efficacy, future expectation, and perceived stress in student-athletes.

## 6 Conclusion

The present study indicates that self-efficacy dimensions are positively associated with several future-expectation domains in high school student-athletes, with social self-efficacy showing the most consistent pattern of associations. Emotional self-efficacy was negatively associated with Perceived Distress in the adjusted model after FDR correction, whereas no self-efficacy dimension remained significantly associated with Perceived Coping. Future-expectation domains were not significantly associated with either perceived-stress component in the adjusted models after FDR correction. Female gender and higher grade level were also associated with greater Perceived Distress. Given the cross-sectional design, these findings should be interpreted as contemporaneous associations rather than as evidence of causal or mediating mechanisms. Longitudinal studies are needed to clarify the temporal relationships among self-efficacy, future expectation, and perceived stress in student-athletes.

## Data Availability

The original contributions presented in the study are included in the article/supplementary material, further inquiries can be directed to the corresponding authors.
